# Imaging Live Bee Brains using Minimally-Invasive Diagnostic Radioentomology

**DOI:** 10.1673/031.012.8901

**Published:** 2012-07-27

**Authors:** Mark K Greco, Jenna Tong, Manucher Soleimani, Duncan Bell, Marc O Schäfer

**Affiliations:** ^1^ Department of Biology and Biochemistry, University of Bath, BA2 7AY, United Kingdom; ^2^ INVERT Centre, Department of Electrical and Electronic Engineering, University of Bath, BA2 7AY, United Kingdom; ^3^ National Reference Laboratory for Bee Diseases, Friedrich-Loeffler-lnstitute, Federal Research Institute for Animal Health, Südufer 10, 17493, Greifswald - Insel Riems, Germany; ^4^ EaSt Anglian Radiography Research, modelling and 3-D printing Group, School of Science, Technology and Health, University Campus Suffolk, Ipswich IP4

**Keywords:** *Apis mellifera*, microCT, X-ray

## Abstract

The sensitivity of the honey bee, *Apis mellifera* L. (Hymeonoptera: Apidae), brain volume and density to behavior (plasticity) makes it a great model for exploring the interactions between experience, behavior, and brain structure. Plasticity in the adult bee brain has been demonstrated in previous experiments. This experiment was conducted to identify the potentials and limitations of MicroCT (micro computed tomograpy) scanning “live” bees as a more comprehensive, non-invasive method for brain morphology and physiology. Bench-top and synchrotron MicroCT were used to scan live bees. For improved tissue differentiation, bees were fed and injected with radiographic contrast. Images of optic lobes, ocelli, antennal lobes, and mushroom bodies were visualized in 2D and 3D rendering modes. Scanning of live bees (for the first time) enabled minimally-invasive imaging of physiological processes such as passage of contrast from gut to haemolymph, and preliminary brain perfusion studies. The use of microCT scanning for studying insects (collectively termed ‘diagnostic radioentomology’, or DR) is increasing. Our results indicate that it is feasible to observe plasticity of the honey bee brain in vivo using diagnostic radioentomology, and that progressive, real-time observations of these changes can be followed in individual live bees. Limitations of live bee scanning, such as movement errors and poor tissue differentiation, were identified; however, there is great potential for in-vivo, non-invasive diagnostic radioentomology imaging of the honey bee for brain morphology and physiology.

## Introduction

The European honey bee, *Apis mellifera* L. (Hymeonoptera: Apidae), workers weigh approximately O.1g. Their brain weighs approximately 0.001 g, has a volume of approximately 1 mm^3^, and has approximately one million neurons (Ribi et al. 2008). The main parts of the brain are the optic lobes, the antennal lobes, the mushroom bodies, and the central complex. The optic and antennal lobes are responsible for processing vision and olfaction, respectively. The mushroom bodies and the central complex constitute the most important centers for behavior, instinct, and memory (Hourcade et al. 2010). Other parts of the brain include the suboesophageal ganglion, tritocerebrum, and ventral cord. It is thought that complex behavior is based on overarching brain networks superimposed on smaller local networks controlling individual responses. Since simple environmental manipulations can both accelerate and delay brain growth in young bees, and since brain volume is sensitive to behavior throughout life, the honey bee has great potential as a model for exploring the interactions between environment, behavior, and brain structure. Experience related changes in brain structure are believed to be an important part of the memory engram (Kolb and Whishaw 1998; Kim and Diamond 2002; Mohammed et al. 2002; Gerber et al. 2004; Kim et al. 2006; Liston et al. 2006), and understanding the relationships between experience and brain structure is key to understanding the relationships between brain and behavior (Kolb and Whishaw 1998). A worker honey bee's natural behavioral change is associated with conspicuous growth of the mushroom bodies in the brain (Withers et al. 1993; Farris et al. 2001; Ismail et al. 2006). The mushroom body calyx is larger in forager bees than same-aged nurse bees that have not left the hive (Withers et al. 1993; Farris et al. 2001). This structural change may be part of the memory engram for the many foraging-related and navigational tasks learned by a forager bee (Farris et al. 2001; Fahrbach et al. 2003).

Phenotypic plasticity in the adult bee brain has been demonstrated in previous experiments using various techniques, such as the Cavalieri or computer volume segmentation methods (Gunderssen & Jenson 1987; Michel & Cruz-Orive 1988; Withers et al. 1993; Brown et al. 2000; Ribi et al. 2008; Maleszka et al. 2009). In all cases, dead bees were used to collect data, which invariably led to differences among individuals.

Our experiment was conducted to identify limitations and potentials for micro computed tomography (MicroCT) scanning of live bees to be used as a comprehensive, non-invasive method for studying brain plasticity, and for teaching morphology and physiology of the brain.

## Materials and Methods

The SYRMEP beamline facilities at the ELETTRA synchrotron in Trieste, and a SCANCO *µ*CT40 bench-top scanner at the University of Bern, were used to scan the bees. At the beamline, newly emerged, adult bees were scanned once daily over five days to observe differential brain plasticity as a result of asymmetric environmental stimuli. Scans on live bees at the beamline facility were performed using phase contrast with the following parameters: 15keV X-ray energy, 20 cm sample to detector distance, a number of projection (over 180°) of 1800, 9 *µ*m isotropic voxel size, 0.9 seconds exposure time, 1 hour 48 minutes measurement time.

**Figure 1.**
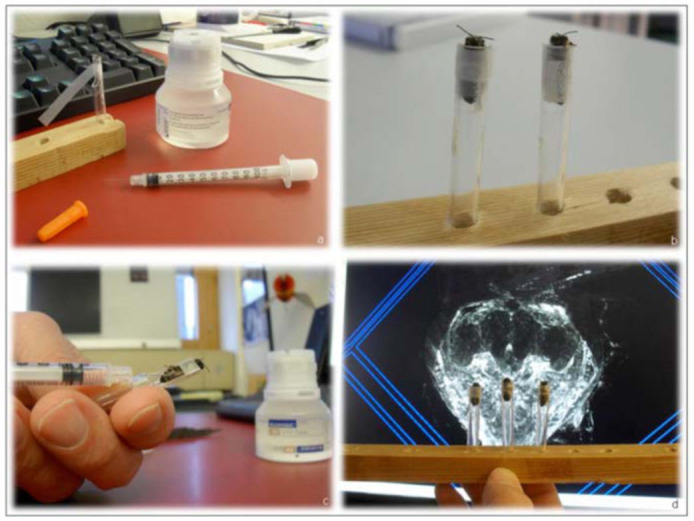
To enhance brain tissue differentiation, bolus injections of radiographic contrast media were delivered via a 3OG needle (a) directly into the haemolymph, between the dorsal abdominal terga, of live bees that were previously secured for scanning (b and c). The 3D rendered brain (d) showed that contrast had perfused into tissue to enable improved structural differentiation. High quality figures are available online.

To enhance tissue differentiation, bolus injections of radiographic contrast media were delivered directly into the haemolymph, between the dorsal abdominal terga, via a 3OG needle (Figure 1). For visual comparisons of gross anatomical features, MicroCT scans of an ancient bee trapped in amber were also performed on the benchtop scanner, using absorption techniques. The tube operating conditions consisted of an HV peak set at 45kV, and a 177*µ*A current. The rest of the parameters were: high resolution mode (1000 Projections/180^°^), 2048 × 2048 pixels image matrix, 10*µ*m size isotropic voxel, 3 seconds integration time, 610 total slices, 2 hours and 30 minutes measurement time.

**Figure 2.**
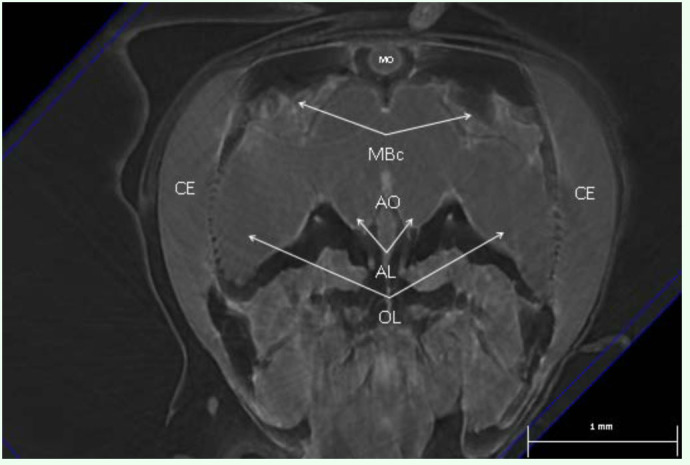
A 3D volume rendered image of a live honey bee's head capsule showing gross morphological structures such as the optic lobes (OL), antennal lobes (AL), aorta (AO), mushroom body calyces (MBc), and median ocellus (MO). The compound eyes (CE) are visualized immediately adjacent and lateral to the optic lobes. High quality figures are available online.

Images and brain volume data (Figure 2) were measured using BeeView volume rendering software (DISECT Systems Ltd).

## Results

**Figure 3.**
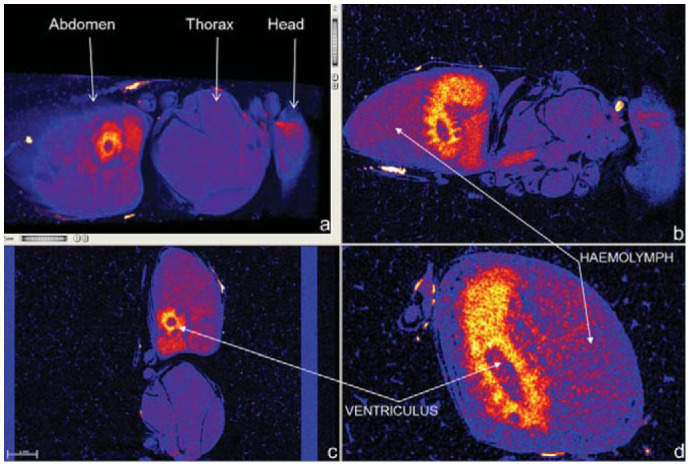
A 3D volume rendered image with BeeView software of a live honey bee showing the three body segments (a) and orthogonal, 2D images (b, c, and d) showing the passage of radiographic contrast from the ventriculus (true stomach) to the haemolymph in the coelum. Images were rendered 1.5 hours after ingestion of contrast. High quality figures are available online.

**Figure 4.**
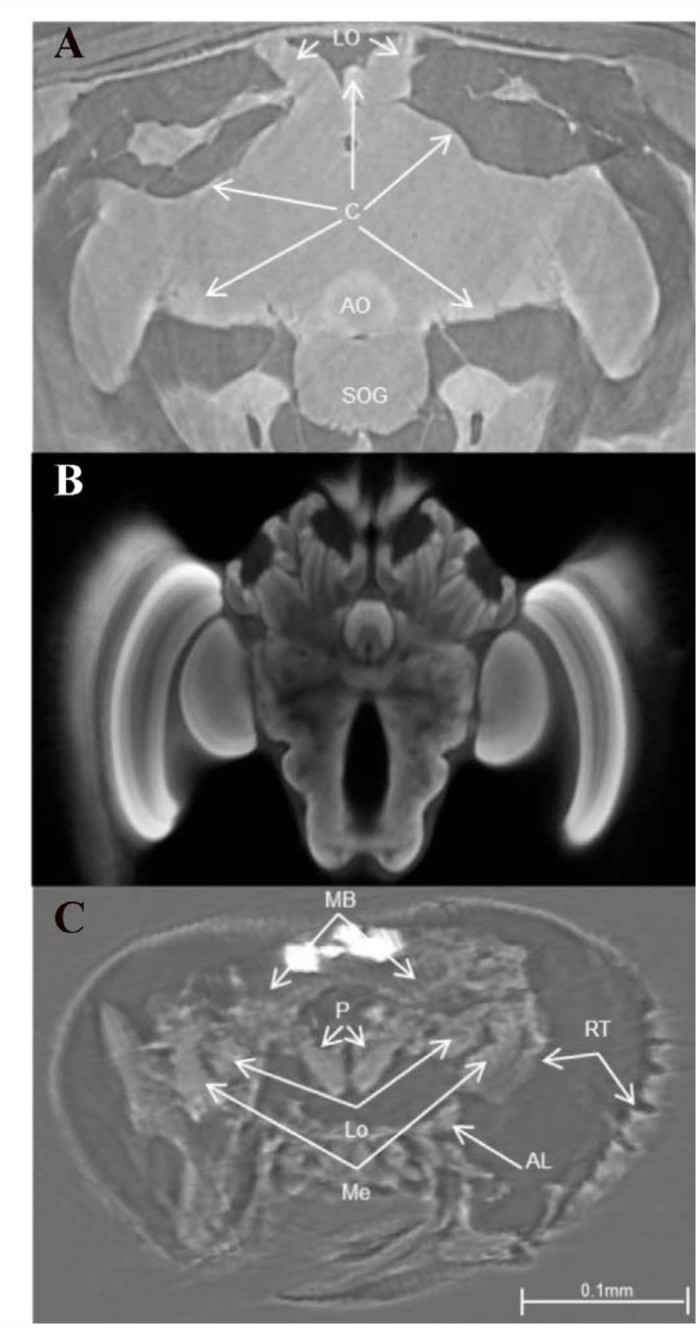
(a) A 2D axial view of a live honey bee brain showing perfusion of contrast medium (C) into peripheral regions. Arrows indicate areas of higher concentration. At 30 minutes post bolus injection into the haemolymph, the lateral ocelli (LO) and aorta (AO) contained more contrast than the sub oesophageal ganglion (SOG), (b) A comparative 2D axial view from the bee brain atlas (http://www.neurobiologie.fu-berlin.de/beebrain/Default.html) that was reconstructed from imaging data from two-channel confocal microscopy scans, (c) An axial view of the head capsule of an ancient stingless bee *Proplebeia abdita* (Greco at al. 2011) trapped in amber. The brain of this 20 million years old bee was particularly well preserved, as evidenced by the optic lobes including the medullae (Me) and lobulae (Lo), antennal lobes (AL), protocerebral lobes (P), and the mushroom bodies (MB). The retinal zone (RT) was also well preserved. High quality figures are available online.

Gross brain morphology, such as the optic lobes, antennal lobes, aorta, mushroom body calyces, and median ocellus, were visualized in 2D and 3D projections. Brain volume measurements (Figure 2) enabled estimates of plasticity. Scanning of live bees enabled minimally-invasive imaging of physiological processes (for the first time), such as passage of contrast from gut to haemolymph (Figure 3), as well as preliminary brain perfusion and plasticity studies (Figure 4a). The image in Figure 4b shows a similar view to Figure 4a, which was produced by Rybak et al. (2010) using data from two-channel confocal microscopy scans. Comparisons of brain images from live extant bees and the 20 million year old bee *Proplebeia abdita* showed little variation in gross morphological features (Figure 4c).

## Discussion

The use of MacroCT and MicroCT imaging for the non-invasive study of insects, collectively termed ‘diagnostic radioentomology’ (DR), is increasing (Hornschemeyer et al. 2002; Johnson et al. 2004; Hönnickea et al. 2005; Greco et al. 2005; Greco et al. 2006; Greco et al. 2008; Greco et al. 2009, Greco et al. 2011). Results from this study indicate that it is feasible to observe plasticity of the honey bee brain ‘in vivo’ using DR, and that progressive, real-time observations of these changes can be followed in individual live bees in association with environmental stimuli. Plasticity in the adult bee brain has been demonstrated in previous experiments using various techniques, such as the Cavalieri or computer volume segmentation methods. In all cases previous to this study, dead bees were used. However, the use of ex-vivo samples increases the chances of fundamental errors in correlation data analyses due to inherent differences among individuals. Movement errors were not a major limitation of this study, because it was possible to completely immobilize the head. However, haemolymph flow continued, which caused exposure variations between tomographic slices. The exposure variations were easily corrected by using the intensity averaging function during image reconstruction. The greatest challenge for this study was achieving adequate brain tissue differentiation, and it was clear that although radiographic contrast showed promise for improving tissue visualization, further improvements on reconstruction algorithms are required to better separate brain structures. Bee brain imaging studies from Ribi et al. 2008 and Rybak et al. 2010 are still of superior quality; however, the results in this experiment demonstrate great potential for in-vivo, non-invasive DR imaging of the honey bee for future research in brain plasticity, and for teaching brain morphology and physiology.

## Abbreviations

DR,: diagnostic radioentomology;
MicroCT,: micro computed tomography
